# Grey hybrid normalization with period based entropy weighting and relational analysis for cities rankings

**DOI:** 10.1038/s41598-023-40954-4

**Published:** 2023-08-23

**Authors:** Moses Olabhele Esangbedo, Jieyun Wei

**Affiliations:** School of Management Engineering, Xuzhou University of Technology, No. 2 Lishui Road, Yunlong District, Xuzhou, 221018 China

**Keywords:** Applied mathematics, Computational science

## Abstract

Researchers have addressed uncertainty in multicriteria decision making from the perspective of the performance values of the alternatives, weighting of the evaluation criteria, and the evaluation methods. Still, they are yet to address the uncertainty caused by the normalization approach. In this paper, we show that different normalization methods, namely sum normalization, min–max normalization, vector normalization, and maximization normalization, can result in different rankings of the alternatives while the performance values and weights are unchanged. We applied the grey system theory to address the problem of uncertainty in this study from three aspects: alternative performance values measurement, criteria weighting, and decision matrix/table normalization within a period. The grey hybrid normalization method is proposed as the main contribution in this paper. Then, we present the rankings of 48 cities under uncertainty to decide the location of a branch office of a Chinese electric vehicle manufacturer as a practical example based on the grey entropy weighting method and grey relational analysis with positive and negative references (GRA-PNR) within the period from the year 2019 to 2021. The research results using this approach ranked New York City the best, with a stock market capitalization of economy validity as the top contributor in terms of weighting. Finally, we used simple additive weighting with grey value and the technique for order of preference by similarity to ideal solution with grey value methods to validate the study results.

## Introduction

The location of a business is one factor that affects its profitability based on its environment. A business environment is the external factors outside the control of the business, such as market size, government policy, and political environment^1^. Since 2020, the coronavirus (COVID-19) pandemic has affected business operations worldwide, but China’s businesses have performed comparatively better until the beginning of 2022^2^. Due to mutation, the virus has developed into various strains that render the already developed vaccine less effective, forcing the government to implement multiple pandemic control measures to save the lives of the Chinese people but put the bottom lines of businesses at risk. However, the survival of businesses equally affects the quality of lives of the employees, so one approach is to establish a business hub as a backup to remain in operation. Thus, alternative locations in a city outside China are needed to continue business operations.

Ranking cities is one approach for location selection. Indices for ranking cities around the world have been established. For example, the Global Cities Index by the American journal Foreign Policy^3^, cities rankings by the Globalization and World Cities Research Network^4^, Global City Competitiveness Index by The Economist Group^5^, Global Cities Initiative rankings by Brookings Institution, and Global Power City Index (GPCI) by The Mori Memorial Foundation^6^. In this study, we chose to rank cities as proxies for the selection of a new location. However, we used secondary data because of the difficulty in obtaining global data. These data are those reported by the GPCI, which reports the performance of 48 cities based on 70 indicators.

Multicriteria decision making (MCDM) is a structured approach to choose the most suitable alternative by considering the importance of the criteria for evaluation and the performance measurements of the alternatives on every criterion^7^. The various levels of importance of the criteria are described by assigning weights to them.

The weight of criteria can change over time; for example, the need for online meetings skyrocketed in the midst of the pandemic, reducing the weight that should be assigned to attending business trips and conferences. Uncertainty exists in weighting because weights can change over a period, and this is represented as grey numbers. Additionally, a grey system is a real system with incomplete or partial information. Deng^8^ proposed grey system theory, and presented the grey relational analysis (GRA) as an MCDM method. Since then, many MCDM approaches have been designed to improve efficacy through combination with other MCDM methods. Furthermore, in this study, we applied the GRA with positive and negative references proposed by Esangbedo et al.^9^ to rank the cities.

The MCDM compensatory procedures for evaluating alternatives involve constructing a decision matrix, normalizing the decision matrix, computing the weighted normalized matrix, and then ranking the alternative based on the MCDM method. The drawback of this method is that different normalization techniques can result in various rankings. Whereas an MCDM ranking result may report the procedure, the decision makers’ (DMs’) desired alternative rankings can be increased based on the normalization method, which should not be the case. A decision matrix *D* has elements $(d_{ij})_{m\times n}$ of the performance value of the $i^\text {th}$ alternative based on the $j^\text {th}$ criterion, where *m* and *n* are the number of alternatives and criteria, respectively; i.e , $1 \le i \le m$ and $1 \le j \le n$. According to Liao and Wu^10^ with Chen^11^, the main types of normalization are:

1. Sum normalization (SN)
  - Beneficial criteria
    1 $$\begin{aligned} d^{\alpha }_{ij}=\frac{d_{ij}}{\sum _{i=1}^{m}d_{ij}} \end{aligned}$$
  - Nonbeneficial criteria
    2 $$\begin{aligned} d^{\alpha \prime }_{ij}=1-\frac{d_{ij}}{\sum _{i=1}^{m}d_{ij}} \end{aligned}$$
2. Minimization to maximization (min–max) normalization (MMN)
  - Beneficial criteria
    3 $$\begin{aligned} d^\beta _{ij}=\frac{d_{ij}- \min \limits _{ i } d_{ij} }{\max \limits _{i} d_{ij}- \min \limits _{i} d_{ij} } \end{aligned}$$
  - Nonbeneficial criteria
    4 $$\begin{aligned} d^{\beta \prime }_{ij}=\frac{\max \limits _{i} d_{ij}- d_{ij} }{\max \limits _{i} d_{ij}- \min \limits _{i} d_{ij} } \end{aligned}$$
3. Vector normalization (VN)
  5 $$\begin{aligned} d^\vartheta _{ij}=\frac{ d_{ij} }{\sqrt{\sum _{i=1}^{m}d_{ij}^2} } \end{aligned}$$

Another type of normalization used by researchers is maximization normalization^12^:

4. Maximization normalization (MN)
  - Beneficial criteria
    6 $$\begin{aligned} d^\eta _{ij}=\frac{d_{ij}}{\max \limits _id_{ij}}=1-\frac{\min \limits _i d_{ij}}{d_{ij}} \end{aligned}$$
  - Nonbeneficial criteria
    7 $$\begin{aligned} d^{\eta \prime }_{ij}=\frac{\min \limits _i d_{ij}}{d_{ij}}=1-\frac{d_{ij}}{\max \limits _i d_{ij}} \end{aligned}$$

**Figure 1:**
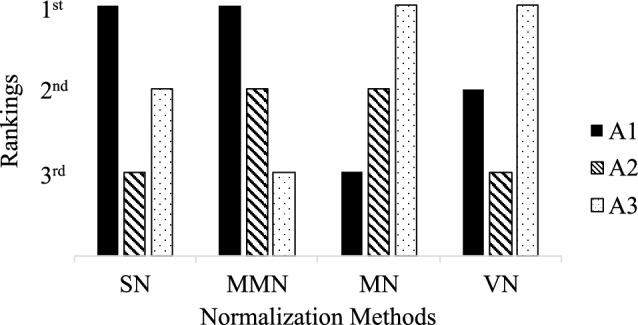
Rankings of the alternatives in Eq. (10).

For simplicity, the equal weighting (EW) method can be defined as

8 $$\begin{aligned} w_j = \frac{1}{m} \end{aligned}$$

and the weighted sum model (WSM) that scores alternative $a_i$ is

9 $$\begin{aligned} a_i = \sum _{1=j}^{n}w_jd^*_{ij}, \end{aligned}$$

where $d^* _{ij}$ is the element of the normalized decision matrix. For example, consider a decision matrix $A_{3X3}$ with three criteria and three alternative that is ranked after using different normalization.

10 $$\begin{aligned} A= \begin{array}{lll} \begin{array}{lll} \quad {{c}_{1}}\quad &{} {{c}_{2}}\quad &{} {{c}_{3}} \\ \end{array} &{} {} \\ \left( \begin{array}{lll} 1 \quad &{} 4\quad &{} 0.6 \\ \!1.5\quad &{} 2\quad &{} \,1 \\ 2\quad &{} 1\quad &{} 1.1 \\ \end{array} \right) &{} \begin{array}{lll} {{a}_{1}} \\ {{a}_{2}} \\ {{a}_{3}} \\ \end{array} \\ \end{array} \end{aligned}$$

using EW ($w_j=1/3$) based on the WSM, the rankings for these three alternatives are: $a_1> a_3 > a_2$ using SN; $a_1> a_2 > a_3$ using MMN, $a_3> a_2 > a_1$ using VN, and $a_3> a_1 > a_2$ using MN. These ranking are shown in Fig. 1.

**Figure 2:**
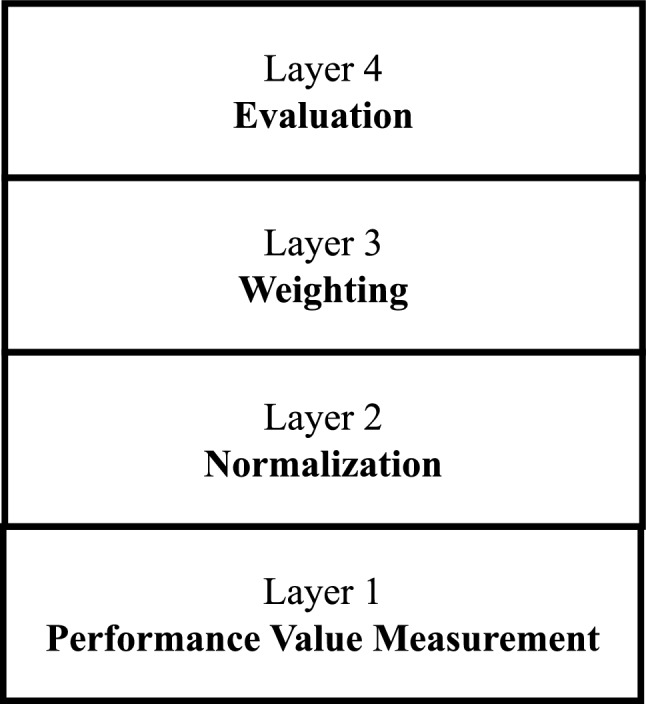
Layers of uncertainties in compensatory MCDM methods.

This a problem because the four different normalization approaches affect the rankings, which is what we addressed in this study. Figure 2 shows layers in MCDM processes where uncertainty can occur. After taking the tally of the rankings as shown in Table 1, neither can we conclude that the first alternative ($a_1$) is better than the third alternative ($a_3$) nor the third alternative ($A_3$) is better than the first alternative ($A_1$).

**Table 1:** Tally of rankings based on the SN, MMN, VN, and MN.

Rankings	$a_1$	$a_2$	$a_3$
$1\text {st}$	2	0	2
$2\text {nd}$	1	2	1
$3\text {rd}$	1	2	1

This uncertainty in ranking caused by the normalization method is addressed using the grey system theory^11^.

The study is motivated by the pursuit of an EV company that wants to control the shock caused by a pandemic by selecting the best location to diversify its operation. Also, this study provides the following contributions: First, we combined four normalization techniques: SN, MMN, VN, and MN, by representing them as grey numbers that account for the uncertainty in decision-making, and we designed a grey hybrid normalization (GHN) approach. Second, we proposed time as an additional dimension in evaluation when considering different snapshots of the performance values of the alternatives over a period. Third, we extended the grey entropy weighing method, called the period-based grey entropy weighting method, over a period to account for uncertainty as time passes by. Another contribution is applying the grey relational analysis with positive and negative (GRA-PNR) in ranking cities as a location selection MCDM problem. To reiterate, the novelty in this paper is the grey hybrid normalization addressing the Layer 2 problem in MCDM. The rest of the paper is organized as follows: “Literature Review” Section presents a literature review. “Methods” Section presents the methods used in this study. “Results and Analysis” Section presents the results and an analysis of our findings. Lastly, Section 5 is the conclusions, which highlights some managerial implications, limitations of this study, and recommendations for future work.

## Literature review

An overview of the MCDM applications for site selection problems can be obtained from Zolfani et al.^13^. Also, a generalized framework for selecting multicriteria methods can be obtained from Watrobski et al.^14^. Researchers have reported the use of MCDM for location selection problems, and in supply chain and logistics management, waste management, and manufacturing and production facility location.

Uncertainties exist in the supply chain and in logistics from retail centers to container hubs. Zhang et al.^15^ analyzed 38 cities to locate multimodal container hubs using grey area relational analysis, and the technique for order of preference by similarity to ideal solution (TOPSIS). They^16^ then evaluated 22 cities as possible locations to serve as Chinese international container hubs by applying GRA and the TOPSIS method. Although vector normalization is commonly used with the classical TOPSIS method, they applied the sum normalization approach. They did not account for uncertainty. However, Wang et al.^17^ developed complex Pythagorean with rough set theory for the location selection of a logistics town project. Yazdani et al.^18^ combined DEA, full consistency method, and combined compromise with the rough set theory as an integrated solution to address the uncertainties in selecting a logistics center location. Yildiz^19^ combined hesitant fuzzy linguistic set with GRA and analytic hierarchy process (AHP) to choose the best location for food retailing in the Turkish food industry. Mahtab et al.^20^ applied an optimization approach in selecting the location of a relief goods distribution facility. Some of these researchers employed grey relational analysis but did not use the grey numbers that can account for uncertainty.

The pandemic has increased the focus on the management of waste. The best worst method (BWM) provides lesser pairwise comparison than the AHP^21^. Torkayesh^22^ combined the BWM and measurement of alternatives and ranking according to co-optimization solution (MARCOS) for selecting a landfill for medical waste. A geographic information System (GIS), BWM, and MARCOS method under grey interval were used in this study to evaluate sustainability factors, and the sum normalization method was employed in the evaluation process. Similarly, Tirkolaee et al.^23^ applied stratified BWM with MARCOS and combined compromise solution (CoCoSo) using grey interval numbers to select healthcare landfill locations, and the MMN method was used. Additionally, Khanlari et al.^24^ used MMN with an optimization function to determine the best location for a temporary hospital, but uncertainty was not considered.

Waste management is not limited to healthcare systems. Pamucar et al.^25^ combined fuzzy set theory (FST) with the measuring attractiveness by a categorical-based evaluation technique (MACBETH) and weight aggregated sum product assessment (WASPAS) method for the selection of a battery recovery center. They used sum normalization in the assessment. Karagoz et al.^26^ applied the SN method with the additive ratio assessment (ARAS) method to select the the location for an end-of-life vehicle recycling facility. The weights of criteria are the mean of the DM points in representing type 2 trapezoidal fuzzy numbers (FNs).

Energy is needed to do work, and the world is shifting to sustainable energy solution. The sum normalization with white numbers for site selection of distributed photo-voltaic (PV) power station by Li et al.^12^. A hybrid of the Entropy weighting method with GRA and TOPSIS was applied in the selection. The TOPSIS and *n*-intuitionistic polygonal fuzzy hybrid aggregation was used to select the electric vehicle (EV) charging station location by Geng and Ma^27^. Also, AHP-II sort with regret theory was equally applied by Liang et al.^28^ in selecting electrical vehicle (EV) charging station location. The AHP-II sort is a dual assignment classification model with sorting for evaluation. Supciller et al.^29^ applied the Stepwise Weight Analysis Ratio Assessment (SWARA) and I-GRA and Evaluation Based on Distance from Average Solution (EDAS) to select the best location for wind farm. The hybrid method used the SN. Conversely, the MMN method in conjunction with the BWM, VlseKriterijuska Optimizacija I Komoromisno Resenje (VIKOR) and GRA to evaluate solar site location by Kannan et al.^30^. Unfortunately, their study used white numbers which does not capture uncertainty in the evaluation. The same is true with Mulliner et al.^31^, that used white numbers with the complex proportional assessment of alternatives (COPRAS) method to evaluate sustainable housing locations. Recently, Javanmardi et al.^32^ present the challenges of uncertainty in sustainability based on GST. Sonar et al.^33^ applied the decision making trial and evaluation laboratory approach to examine the factors for EV purchase and discovered charging time, driving range and price as the primary factors affecting EV adoption. Tavana et al.^34^ used the Internet of Things and big data to design a sustainable supply chain model for EV battery production-a fuzzy bi-objective mixed-integer linear programming model for manufacturing, remanufacturing, and distributing EV lithium-ion batteries.

FST is used in the selection of location, and the SN is commonly used. Nazari et al.^35^ applied the SN with the AHP for the selection of landfill site. Also, Kuo et al.^36^ and Kahraman et al.^37^ applied the SN with the mean of the DMs points with the AHP for convenience store location and motor factory location, respectively. Ertugrul and Karakasoglu^38^ used SN with the fuzzy AHP TOPSIS method in the selection of the location of a textile company in Turkey. Similarly, Cebi and Otay^39^ the SN with the DM’s preferences as represented by FNs, and the TOPSIS in the cement factory location. Unlike other researchers, Yong et al.^40^ applied VN with the TOPSIS method to evaluate plant location. They used the fuzzy point allocation (PA) method to determine the weights of the evaluation criteria. Similarly, Paul^41^ applied the TOPSIS method in the selection of manufacturing plant locations, where the fuzzy mean of the DMs point was used for weighting. Stanujkic et al.^42^ employed an optimization approach in the selection of production plant locations.

Location-selection MCDM approaches require no normalization, which is common when all criteria are on the same scale. For example, Tadic^43^ applied Delph, AHP, and CODAS using grey numbers (GN) for the selection of dry port terminal locations without normalization. Wang et al.^17^ applied RN with the Heronian mean aggregation operator to formulate a complex Pythagorean uncertain linguistic rough interaction with the Heronian mean operator for a logistic town selection project. Anvari^44^ evaluated the location of a supply facility in China using the AHP and WSM without normalization. Ma et al.^45^ applied homophily-based relaxation algorithm optimization for the selection of a coffee shop location, which did not need normalization. However, Akyurt et al.^46^ selected the location for the best air flight base training using the measuring attractiveness by a categorical based evaluation technique (MACBETH) and ranking of alternatives through functional mapping of criterion subintervals into a single interval (RAFSI), which used the arithmetic and harmonic mean for normalization.

Aytekin^47^ presented some analysis of the various normalization method. He noted that some normalization methods might result in rank reversal, and highlighted that the type of data in the performance value is a critical factor in the selection process. For example, not all methods can be used with zero or negative performance values. Mukhametzyanov^48^ showed the characteristic of the entropy weighting method is sensitive to the probability of states when compared to the standard deviation and criteria importance through inter-criteria correlation weighting method. Mukhametzyanov^49^ eliminated the domains’ displacement of the normalized values and introduced the IZ-method. Furthermore, he compared the reverse sorting algorithm, IZ-method, and mean and standard deviation method. The IZ-method performed better than classical normalization methods^50^.

There are records of researchers evaluating cities, especially in China. Shi et al.^51^ evaluated green cities from 15 provinces in China by developing an indexing system and using the entropy weighting method in the evaluation. In this study, the max-min normalization method was used. Shen et al.^52^ comprehensively evaluated the performance of smart cities in China, where the Chinese government policy is selecting the evaluation criteria, the entropy weighing method and the TOPSIS method were combined for ranking. Zhou et al.^53^ evaluated the sustainability of the 14 cities in Liaoning, China. Interestingly, this study used the reciprocal value of the performance value to represent cost criteria and then used sum normalization to scale the performance value to unity. The result depicted the eastern Liaoning cities to be more sustainable. Wanqing^54^ evaluated international port cities using fuzzy AHP. Li et al.^12^ presented a smart city shareable framework with criteria such as cloud environment, information resources and security for evaluating 17 cities in China using the WSM. Nakamura and Managi^55^ investigated the effect of objective evaluation consisting of environmental, social, and economic aspects and subjective evaluation consisting of personal municipality service evaluation indicators for life satisfaction in Japan, reviewing that objective city evaluation does not contribute to life satisfaction to the Japanese in most cases since environmental and economic aspect contributes negatively to the life satisfaction. Unlike previous studies, this research is multi-national studies.

A apparent problem with a number of MCDM methods is rank reversal. Žižoviç et al.^56^ developed a ranking of alternatives through functional mapping of criterion sub-intervals into a Single Interval to solve the rank reversal problem, where sub-interval mapping is used to make both beneficial and cost criteria unidirectional. Trung et al.^57^ compared the RAFSI and Proximity Indexed Value, then presented combinations of the evaluation method based on the removal effects of criteria (MEREC), rank order centroid (ROC), rank sum and equal weighting method. Esangbedo and Che^58^ combined the ROC weights with GRA to evaluate businesses in Africa that used the cities in all African countries as proxies in measuring the performance value of nations. A similar study was conducted for west-African countries using cities as proxies in evaluating the countries based on the grey-WSM^59^. Furthermore, Esangbedo et al.^9^ proposed the ROC with slacks for Photothermal Power Station Construction; undoubtedly, upon completion of the station, among its numerous uses, it will be used for charging EVs. Esangbedo and Tang^60^ applied the MEREC and multi-attribute ideal-real comparative analysis based on the grey system theory to evaluate heavy machinery company decolonization systems.

Wang^61^ developed a Malmquist index for the GPCI index, which is different from our study in its contribution. As highlighted in these studies, SN and MMN are predominantly used in normalizing decision matrices and weighting criteria. Also, Liao and Wu^10^ identified the problem with SN and MMN and proposed double normalization-based multiple aggregation method, but their studies did not capture the four layers of uncertainties in Fig. 2. After searching the literature, to the best of our knowledge, this is the first study highlighting the effects of normalization in MCDM methods with a simple example (Eq. 10). Additionally, this study fills the gap in the literature by estimating the weight of decision criteria over a period, not just at a specific point in time. Table 2 summarizes the findings from the selected literature reviewed.

**Table 2:** Summary normalization of MCDM location selection.

Normalization methods	Type of numbers	Weighting methods	Evaluation methods	Application	Researchers
MN	WN	BWM	MARCOS	Waste location	Torkayesh et al.^22^
MN	WN	Entropy	Grey relational projection	Distributed PV power station site	Li et al.^12^
–	WN	AHP-II sort	Regret theory	EV Charging station location	Liang et al.^28^
SN	WN	n-IPFHA	TOPSIS	EV Charging Station Location	Geng and Ma^27^
SN	WN	Entropy	GRA-TOPSIS	Container multimodal hubs	Zhang et al.^16^
SN	WN	PA	COPRAS	Sustainable housing	Mulliner et al.^31^
SN	GN	SWARA	I-GRA EDAS	Wind farm location	Supciller and Bayramoglu^29^
SN	GN	Grey Preference Selection Index	Grey Proximity Indexed Value	Warehouse location selection	Ulutas et al.^62^
SN	FN	MACBETH	DWAO, DWGAO WASPAS	Recovery center for batteries	Pamucar et al.^25^
SN	FN	Mean PA	ARAS	End-of-life vehicles recycling facility	Karagoz et al.^26^
SN	FN	Mean PA	TOPSIS	Cement factory location selection	Cebi and Otay^39^
SN	FN	Mean PA	AHP	Convenience store location	Kuo et al.^36^
SN	FN	Mean PA	AHP	Car factory location	Kaharman et al.^37^
SN	FN		AHP	Landfill site selection	Nazari et al.^35^
SN	FN	AHP	TOPSIS	Textile company location	Ertugru^38^
–	RN	MACBETH	RAFSI	Flight Base Selection	AkyurtI et al.^46^
MMN	WN	Risk-averse BWM	Optimization model	Temporary hospital location	Kheybari et al.^63^
MMN	GN	Stratified BWM	Grey CoCoSo	Healthcare waste disposal	Tirkolaee and Torkayesh^23^
MMN	WN	BWM	VIKOR, GRA	Solar sites location	Kannan et al.^30^
MMN	RN	DEA FUCOM	R-CoCoSo	Logistics centers location	Yazdani et al.^18^
MMN			GRA GARA TOPSIS	Logistics hub location	Zhang et al.^15^
MMN	FN	Hesitant AHP	GRA	Food retailing location	Yildiz & Tuysuz^19^
			Stochastics Optimization	Logistic	Mahtab et al.^20^
–	WN	optimization	AHP WSM	Facility location in Supply Chain network	Anvari & Turkay^44^
SN				Facility location	Ertugrul and Karakasoglu^38^
VN	FN	PA	TOPSIS	Plant location	Yong^40^
	FN	Mean of PA	TOPSIS	Manufacturing plant location	Paul et al.^41^
	FN			Production plant location selection	Stanujkic and Kavaliauskiene^64^
–	FN		GRA	Market hall location	Bilisik et al.^65^
–	RN		Optimization	Logistics town project	Wang & Zhang^17^
–	GN	Delphi, AHP	CODAS	Dry port terminal location	Tadic et al.^43^
–	WN		Homophily-based relaxation algorithm optimization	Coffee shop location	Ma et al.^45^

*WN* white numbers, *GN* grey numbers, *FN* fuzzy numbers, *RN* rough numbers

## Methods

### Evaluation criteria

The evaluation criteria we used in this study are those presented by GPCI, which is freely available to the public for download at http://www.mori-m-foundation.or.jp The hierarchical model consists of 6 first-level indicators, 26 second-level indicators, and 70 third-level indicators. A summary and a truncated hierarchical model are presented in Table 3. The criterion *Economic* ($C_1$) is the ability for a business to be highly profitable based on factors such as market size, human capital, and the ease of establishing a business. *Research and Development* ($C_2$) measures the academic resources, such as the number of tertiary institutions, and the level of inventiveness, such as the number of patents and successful startups. *Cultural Interaction* ($C_3$) captures the people’s way of life as it influences nonindigenous people, such as resources for tourism and amenities such as hotels for visitors. *Livability* ($C_4$) measures the ease of settling in the cities, for example, people feeling safe because of security and low likelihood of natural disasters. *Environment* ($C_5$) measures the natural environment, which includes the absence of pollution and the city’s commitment to sustainability. *Accessibility * ($C_6$) measures the availability of road networks and cheap with readily available public transportation.

**Table 3:** Hierarchical model for cities rankings.

First-level criteria ($C_p$)	Second-level criteria ($C_{p-q}$)	Third-level criteria ($C_{p-q-r}$)	Index (*v*)
Economic ($C_1$)	Market Sizie ($C_{1-1}$)	Nominal GDP ($C_{1-1-1}$)	1
		GDP per Capita ($C_{1-1-2}$)	2
	Market Attractiveness ($C_{1-2}$)	GDP Growth Rate ($C_{1-2-1}$)	3
		Economic Freedom ($C_{1-2-2}$)	4
R & D (${C_2}$)	$\vdots$	$\vdots$	$\vdots$
Cultural Interaction (${C_3}$)			
Livability (${C_4}$)			
Environment (${C_5}$)			
Accessility(${C_6}$)	International Network (${C_{6-1}}$)		
	Air Transport Capacity (${C_{6-2}}$)		
	Inter-city Transportation (${C_{6-3}}$)		
	Transport Comfortability (${C_{6-4}}$)	Communiting Time (${C_{6-4-1}}$)	68
		Traffic Congession (${C_{6-4-2}}$)	69
		Ease of Mobility by Taxi or Bycicle (${C_{6-4-3}}$)	70

See the GPCI year 2021 for details indicators with explanations

### Uncertainty in weighting

**Figure 3:**
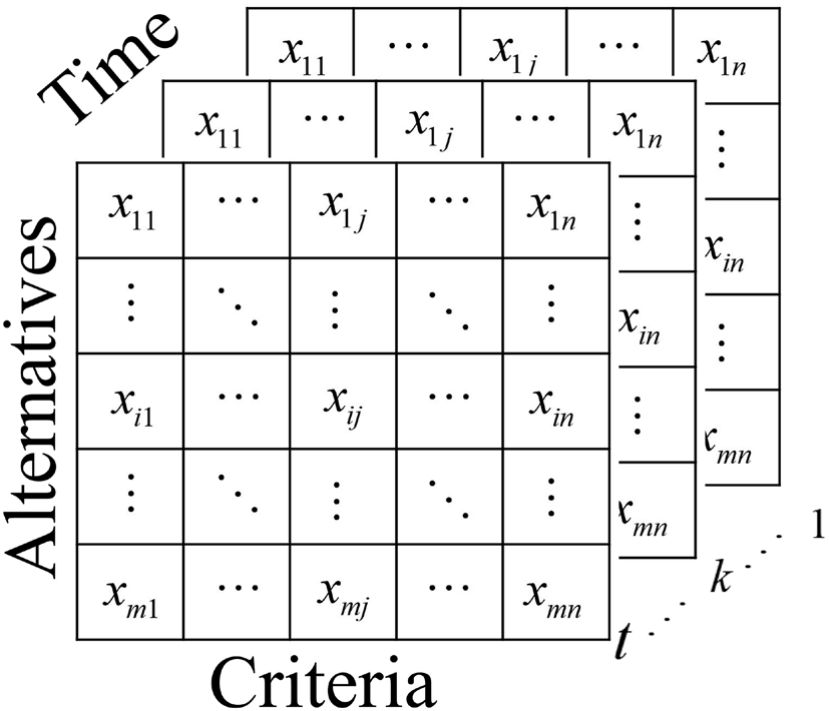
Decision matrix of over a period.

As time passes by snapshot of the performance values of the alternative changes, and so are the entropy weight. Figure 3 shows the decision matrix at various time $(1 \le k \le t)$. Where *k* is the time between the period from 1 to *t*. The entropy weighting method estimates the weights based on the extent the information in the system is reflected and the uncertainty in the system. The entropy weighting method is used to calculate the weights of the criteria at different times, and then these weights for each of the decision criteria are converted into GN by taking the minimum and maximum weight in the period.

For a vector ${{x}_{jk}}=({{x}_{1jk}},{{x}_{2jk}},\ldots ,{{x}_{mjk}})$, the entropy contrast intensity of the *j*th criterion after normalization at time *k* is given as:

11 $$\begin{aligned} {{e}_{jk}}=-\frac{1}{\ln m}\sum \limits _{i=1}^{m}{\frac{{{x}_{ijk}}}{{{X}_{jk}}}\ln \frac{{{x}_{ijk}}}{{{X}_{jk}}}}, \end{aligned}$$

where *X* is a term of the ${{i}{\textrm{th}}}$ criterion at the time *k*,

12 $$\begin{aligned} {{X}_{jk}}=\sum \limits _{i=1}^{m}{{{x}_{ijk}}},\quad j=1,2,\ldots ,n. \end{aligned}$$

The weights at time *k* can be estimated as:

13 $$\begin{aligned} {{w}_{jk}}=\frac{1-{{e}_{jk}}}{\sum \nolimits _{j=1}^{n}{\left( 1-{{e}_{jk}} \right) }}. \end{aligned}$$

Then, the period-based grey entropy weight is given as:

14 $$\begin{aligned} \otimes w_j = [ \underline{w_j},\overline{w_j} ] = [\min \limits _{k}w_{ik}, \max \limits _{k}w_{ik} ] \end{aligned}$$

Recall some basic operations of two-interval grey numbers $\otimes x = [{\underline{x}}, {\overline{x}}]$ and $\otimes y = [{\underline{y}}, {\overline{y}}]$ is a follows^66,67^:

15 $$\begin{aligned} \otimes x + \otimes y= & {} \left[{{\underline{x}} + {\underline{y}},{\overline{x}} + {\overline{y}}} \right]\end{aligned}$$

16 $$\begin{aligned} c \times \otimes x= & {} \left[{c{\underline{x}},c{\overline{x}}} \right]\end{aligned}$$

17 $$\begin{aligned} - c \times \otimes x= & {} \left[{- c{\overline{x}}, - c{\underline{x}}} \right]\end{aligned}$$

18 $$\begin{aligned} \otimes x \times \otimes y=\, & {} \left[{{\underline{x}},{\overline{x}}} \right]\times \left[{{\underline{y}},{\overline{y}}} \right]= \left[{\text {min}\left( {{\underline{x}}{\underline{y}},{\underline{x}}{\overline{y}},{\overline{x}}{\underline{y}},{\overline{x}}{\overline{y}}} \right) ,\text {max}\left( {{\underline{x}}{\underline{y}},{\underline{x}}{\overline{y}},{\overline{x}}{\underline{y}},{\overline{x}}{\overline{y}}} \right) } \right]\end{aligned}$$

19 $$\begin{aligned} \otimes x - \otimes y= & {} \otimes x + \left( {- 1 \times \otimes y} \right) = \left[{{\underline{x}} - {\overline{y}},{\overline{x}} - {\underline{y}}} \right]\end{aligned}$$

20 $$\begin{aligned} \otimes x \div \otimes y= & {} \left[{{\underline{x}},{\overline{x}}} \right]\times \left[{\frac{1}{{\underline{y}}},\frac{1}{{\overline{y}}}} \right]. \end{aligned}$$

21 $$\begin{aligned} \left| {\otimes x - \otimes y} \right|= & {} \text {max}\left( {\left| {{\underline{x}} - {\underline{y}}} \right| ,\left| {{\overline{x}} - {\overline{y}}} \right| } \right) . \end{aligned}$$

Equation (24) is an arbitrary distance between $\otimes x$ and $\otimes y$.

This weight can be used with any MCDM evaluation method, and is used in subsequent sections.

### Uncertainty in performance value

At different times, the performance of the alternative is different in a dynamic decision-making problem. This uncertainty is represented as a grey number by measuring the minimum and maximum values of the performance value over a period. A decision matrix *X* at time *k* can be represented as:

22 $$\begin{aligned} X_k = \left( \begin{matrix} {{x}_{11k}} &{}\dots &{} {{x}_{1jk}} &{}\dots &{} {{x}_{1nk}} \\ \vdots &{}\ddots &{}\vdots &{} \ddots &{}\vdots \\ {{x}_{i1k}} &{} \dots &{}{{x}_{ijk}} &{}\dots &{}{{x}_{ink}} \\ \vdots &{}\ddots &{}\vdots &{}\ddots &{}\vdots \\ {{x}_{m1k}} &{} \dots &{}{{x}_{mjk}} &{} \dots &{}{{x}_{mnk}} \\ \end{matrix} \right) , \end{aligned}$$

and the grey decision matrix is

23 $$\begin{aligned} \otimes X_ = \left( \begin{matrix} {{\otimes x}_{11}} &{}\dots &{} {{\otimes x}_{1j}} &{}\dots &{} {{\otimes x}_{1n}} \\ \vdots &{}\ddots &{}\vdots &{} \ddots &{}\vdots \\ {{\otimes x}_{i1}} &{}\dots &{}{{\otimes x}_{ij}} &{}\dots &{}{{\otimes x}_{in}} \\ \vdots &{}\ddots &{}\vdots &{}\ddots &{}\vdots \\ {{\otimes x}_{m1}} &{} \dots &{}{{\otimes x}_{mj}} &{} \dots &{}{{\otimes x}_{mn}} \\ \end{matrix} \right) , \end{aligned}$$

where $$\otimes x_{ij} =[ \underline{x_{ij}}, \overline{x_{ij}}]= [\min \limits _{k}x_{ijk}, \max \limits _{k}x_{ijk} ]$$

### Uncertainty in normalizing decision table

The uncertainty in normalization is addressed by using SN, MMN, and VN, based on GST. The normalization of grey numbers is as follows:

1. Grey sum normalization (GSN)^68^
  - Beneficial criteria
    24 $$\begin{aligned} \begin{aligned} \otimes d^\alpha _{ij} =&[\underline{d^\alpha _{ij}}, \overline{d^\alpha _{ij}}]\\ =&\left[ \frac{\underline{d_{ij}}}{\sum _{i=1}^{m} \overline{d_{ij}}}, \frac{\overline{d_{ij}}}{\sum _{i=1}^{m} \overline{d_{ij}}}\right] , \end{aligned} \end{aligned}$$
  - Nonbeneficial criteria
    25 $$\begin{aligned} \begin{aligned} \otimes d^{\alpha \prime }_{ij} =&[\underline{d^{\alpha \prime }_{ij}}, \overline{d^{\alpha \prime }_{ij}}]\\ =&\left[ 1-\frac{\overline{d_{ij}}}{\sum _{i=1}^{m} \overline{d_{ij}}}, 1-\frac{\underline{d_{ij}}}{\sum _{i=1}^{m} \overline{d_{ij}}}\right] . \end{aligned} \end{aligned}$$
2. Grey min–max normalization (GMMN)^69^
  - Beneficial criteria
    26 $$\begin{aligned} \otimes d^{\beta } ij & = [\underline{{d_{{ij}}^{\beta } }} ,\overline{{d_{{ij}}^{\beta } }} ] \\ & = \left[ {\frac{{\underline{{d_{{ij}} }} - \mathop {\min }\limits_{i} \underline{{d_{{ij}} }} }}{{\mathop {\max }\limits_{i} \overline{{d_{{ij}} }} - \mathop {\min }\limits_{i} \underline{{d_{{ij}} }} }},\frac{{\overline{{d_{{ij}} }} - \mathop {\min }\limits_{i} \underline{{d_{{ij}} }} }}{{\mathop {\max }\limits_{i} \overline{{d_{{ij}} }} - \mathop {\min }\limits_{i} \underline{{d_{{ij}} }} }}} \right], \\ \end{aligned}$$
  - Nonbeneficial criteria
    27 $$\begin{aligned} \otimes d_{{ij}}^{{\beta ^{\prime } }} & = [\underline{{d_{{ij}}^{{\beta ^{\prime } }} }} ,\overline{{d_{{ij}}^{{\beta ^{\prime } }} }} ] \\ & = \left[ {\frac{{\mathop {\max }\limits_{i} \overline{{d_{{ij}} }} - \overline{{d_{{ij}} }} }}{{\mathop {\max }\limits_{i} \overline{{d_{{ij}} }} - \mathop {\min }\limits_{i} \underline{{d_{{ij}} }} }},\frac{{\mathop {\max }\limits_{i} \overline{{d_{{ij}} }} - \underline{{d_{{ij}} }} }}{{\mathop {\max }\limits_{i} \overline{{d_{{ij}} }} - \mathop {\min }\limits_{i} \underline{{d_{{ij}} }} }}} \right]. \\ \end{aligned}$$
3. Grey vector normalization (GVN), deductively:
  28 $$\begin{aligned} \begin{aligned} \otimes d^\vartheta _{ij} =&[\underline{d^\vartheta _{ij}}, \overline{d^\vartheta _{ij}}]\\=&\left[ \frac{ \underline{d_{ij}} }{\sqrt{\sum _{i=1}^{m}\otimes d_{ij}^2} },\frac{ \overline{d_{ij}} }{\sqrt{\sum _{i=1}^{m}\otimes d_{ij}^2} }\right] . \end{aligned} \end{aligned}$$
4. Grey sum normalization (GMN)^68^
  - Beneficial criteria
    29 $$\begin{aligned} \otimes d^\eta _{ij} = [\underline{d^\eta _{ij}}, \overline{d^\eta _{ij}}] =\left[ \frac{\underline{d_{ij}}}{\max \limits _i \overline{d_{ij}}}, \frac{\overline{d_{ij}}}{\max \limits _i \overline{d_{ij}}}\right] , \end{aligned}$$
  - Nonbeneficial criteria
    30 $$\begin{aligned} \begin{aligned} \otimes d^{\eta \prime }_{ij} =&[\underline{d^{\eta \prime }_{ij}}, \overline{d^{\eta \prime }_{ij}}]\\ =&\left[ 1-\frac{\overline{d_{ij}}}{\max \limits _i \overline{d_{ij}}}, 1-\frac{\underline{d_{ij}}}{\max \limits _i \overline{d_{ij}}}\right] . \end{aligned} \end{aligned}$$
5. Grey hybrid normalization (GHN), which is proposed in this paper, is:
  31 $$\begin{aligned} \begin{aligned} \otimes d^{*}_{ij}&= [\underline{d^{*}_{ij}}, \overline{d^{*}_{ij}}]\\ {}&= [\min (\underline{d^{\alpha }_{ij}},\underline{d^{\beta }_{ij}}, \underline{d^{\vartheta }_{ij}}, \underline{d^{\eta }_{ij}}),\max (\overline{d^{\alpha }_{ij}},\overline{d^{\beta }_{ij}}, \overline{d^{\vartheta }_{ij}}, \overline{d^{\eta }_{ij}})]. \end{aligned} \end{aligned}$$
  In other words, GHN is the union of SN, MMN, VN, and MN.
  32 $$\begin{aligned} \otimes d^{*}_{ij} = \otimes d^{\alpha }_{ij}\cup \otimes d^{\beta }_{ij}\cup \otimes d^{\vartheta }_{ij}\cup \otimes d^{\eta }_{ij} \end{aligned}$$

Although the TOPSIS has long been extended using GST to accommodated uncertainty, studies using GVN are scarce. One main possible reason for this is the computational complexity of GVN, because GSN, GMMN, and GMN are easier to compute.

### GRA with positive and negative references

GRA with positive and negative references (PNR) method was proposed by Esangbedo et al.^9^ and addresses the limitation of a single point of reference in comparing two grey numbers. After obtaining the weighted normalized matrix,

33 $$\begin{aligned} \otimes D^\prime = \otimes W \times \otimes D, \end{aligned}$$

the positive reference alternative (PRA) and negative references alternative (NRA) are obtained.

1. PRA (34):
  34 $$\begin{aligned} D_{0}^{+} = \left\{ \otimes d_{01}^{+}, \otimes d_{02}^{+},\ldots , \otimes d_{0n}^{+} \right\} \end{aligned}$$
   where
  $$\begin{aligned} \otimes d_{0j}^{+} = \left[{\underset{1 \le i \le m}{\text {max}}\underline{d_{ij}^{+}},\underset{1 \le i \le m}{\text {max}}\overline{d_{ij}^{+}}} \right]. \end{aligned}$$
2. NRA (35):
  35 $$\begin{aligned} D_{0}^{-} = \left\{ \otimes d_{01}^{-}, \otimes d_{02}^{-},\ldots , \otimes d_{0n}^{-} \right\} \end{aligned}$$
   where
  $$\begin{aligned} \otimes d_{0j}^{-} = \left[{\underset{1 \le i \le m}{\text {min}}\underline{d_{ij}^{-}},\underset{1 \le i \le m}{\text {min}}\overline{d_{ij}^{-}}} \right]. \end{aligned}$$

Then, the difference between the PRA and normalized weighted alternatives, as well as the difference between the normalized weighted alternatives and NRA are computed;

1. Difference between PRA and alternatives is
  36 $$\begin{aligned} { {\Delta } }^+ = \left( \begin{array}{cccc} \delta ^+_{1,1} &{} \delta ^+_{1,2} &{} \cdots &{} \delta ^+_{1,n} \\ \delta ^+_{2,1} &{} \delta ^+_{2,2} &{} \cdots &{} \delta ^+_{2,n} \\ \vdots &{} \vdots &{} \ddots &{} \vdots \\ \delta ^+_{m,1} &{} \delta ^+_{m,2} &{} \cdots &{} \delta ^+_{m,n} \\ \end{array} \right) \end{aligned}$$
  where $$\delta _{ij}^+ = {\overline{d_{0j}^{+}} - \overline{d_{ij}^{*}}}$$
2. Difference between alternatives and NRA is
  37 $$\begin{aligned} { {\Delta } }^- = \left( \begin{array}{cccc} \delta ^-_{1,1} &{} \delta ^-_{1,2} &{} \cdots &{} \delta ^-_{1,n} \\ \delta ^-_{2,1} &{} \delta ^-_{2,2} &{} \cdots &{} \delta ^-_{2,n} \\ \vdots &{} \vdots &{} \ddots &{} \vdots \\ \delta ^-_{m,1} &{} \delta ^-_{m,2} &{} \cdots &{} \delta ^-_{m,n} \\ \end{array} \right) \end{aligned}$$
  where $$\delta _{ij}^-= {\underline{d_{ij}^{*}} - \overline{d_{0j}^{-}}}$$

Next, the positive and negative grey relational grades are computed:

1. Positive grey relational grades:
  38 $$\begin{aligned} r_{i}^+ = \frac{1}{n}{\sum \nolimits _{j = 1}^{n}\gamma _{ij}^+}, \end{aligned}$$
  where the positive grey relational coefficient is:
  $$\begin{aligned} \gamma _{ij}^+ = \frac{\underset{1 \le i \le m}{\text {min}}\underset{1 \le j \le n}{\text {min}}\delta _{ij}^+ + \zeta \underset{1 \le i \le m}{\text {max}}\underset{1 \le j \le n}{\text {max}}\delta _{ij}^+}{\delta _{ij}^+ + \zeta \underset{1 \le i \le m}{\text {max}}\underset{1 \le j \le n}{\text {max}}\delta _{ij}^+}. \end{aligned}$$
2. Negative grey relational grades:
  39 $$\begin{aligned} r_{i}^- = \frac{1}{n}{\sum \nolimits _{j = 1}^{n}\gamma _{ij}^-}, \end{aligned}$$
  where the negative grey relational coefficient is:
  $$\begin{aligned} \gamma _{ij}^- = \frac{\delta _{ij}^- + \zeta \underset{1 \le i \le m}{\text {max}}\underset{1 \le j \le n}{\text {max}}\delta _{ij}^-}{\underset{1 \le i \le m}{\text {min}}\underset{1 \le j \le n}{\text {min}}\delta _{ij}^- + \zeta \underset{1 \le i \le m}{\text {max}}\underset{1 \le j \le n}{\text {max}}\delta _{ij}^-}. \end{aligned}$$
  A distinguishing grey coefficient of 0.5, ($\zeta$ = 0.5), is used^8^.

Lastly, the rank scores are obtained and ranked, which corresponds to the rankings of the alternatives:

40 $$\begin{aligned} V_i = r_i^-\left( 1-\lambda \right) + r_i^+ \lambda \end{aligned}$$

where $\lambda$ is the grey relational grades reference coefficient.

## Results and analysis

### Location selection uncertainty

A Chinese electric vehicle (EV) company needs to open a research and service company, which would act as a branch office in another city.

**Figure 4:**
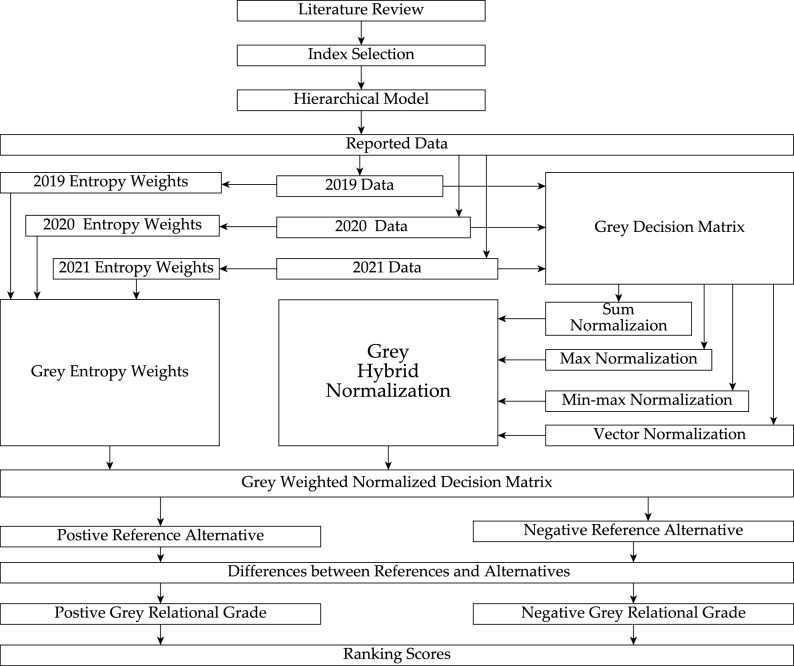
Flowchart of city rankings under uncertainty.

Then, company used the GPCI for evaluation; however, the top management were dissatisfied because the index does not capture uncertainty, which is crucial considering the COVID-19 pandemic. Moreover, the GPCI uses equal weights for the second-level criteria. Every decision maker knows that for nongeneric decision-making problem, all criteria should not have equal weights. A team was summoned to incorporate uncertainty to the GPCI. Thus, the need arose to compute the weight of the criteria under uncertainty to capture the dynamic nature of a city being evaluated. Based on the method presented “Methods” Section, the flowchart for location selection is given in Fig. 4. In addressing this problem, we evaluated 48 cities based on six first-level criteria. The period-based decision matrix in Fig. 3 is represented a period-based decision table, as shown in Table 3, and we used the decision table in Table 4 to construct the decision matrix.

**Table 4:** Decision table.

Time (*k*)	$A_i$	Indicator (*v*) cities	1	2	3	$\dots$	70
2019	$A_1$	Amsterdam	7.1	49.7	32.9	$\dots$	39.3
	$A_2$	Bangkok	14.4	7.4	37.4	$\dots$	96.6
	$A_3$	Barcelona	7.8	19.6	4.4	$\dots$	58.2
	$A_4$	Beijing	50.6	8.4	90.5	$\dots$	93.4
	$A_5$	Berlin	15.1	20.6	27.7	$\dots$	34.3
	$A_6$	Boston	7.6	44.7	30.1	$\dots$	59.6
	$\vdots$	$\vdots$	$\vdots$	$\vdots$	$\vdots$	$\ddots$	$\vdots$
	$A_{48}$	Zurich	5.6	87	25.3	$\dots$	0
2020	$A_1$	Amsterdam	8	53	38	$\dots$	100
	$A_2$	Bangkok	15.8	8.1	35.8	$\dots$	56.5
	$A_3$	Barcelona	8.1	27.9	27.2	$\dots$	53.7
	$A_4$	Beijing	53.4	9.3	79.6	$\dots$	95.4
	$A_5$	Berlin	16.6	22.2	44.4	$\dots$	51.1
	$A_6$	Boston	7.8	45.5	33.1	$\dots$	45.4
	$\vdots$	$\vdots$	$\vdots$	$\vdots$	$\vdots$	$\ddots$	$\vdots$
	$A_{48}$	Zurich	5.7	82.3	18.8	$\dots$	31
2021	$A_1$	Amsterdam	7.1	53.8	49.2	$\dots$	100
	$A_2$	Bangkok	15.5	9.1	51.8	$\dots$	56.5
	$A_3$	Barcelona	7.2	28.2	50.7	$\dots$	53.7
	$A_4$	Beijing	46.9	10.7	85.7	$\dots$	95.4
	$A_5$	Berlin	14.5	22.3	55.2	$\dots$	51.1
	$A_6$	Boston	7.3	47.5	48	$\dots$	45.4
	$\vdots$	$\vdots$	$\vdots$	$\vdots$	$\vdots$	$\ddots$	$\vdots$
	$A_{48}$	Zurich	5.5	82.3	42.8	$\dots$	31

See the GCPI yearbook 2019, 2020, and 2021

Based on Eq. (13), the decision matrix for the year 2019 is :

41 $$\begin{aligned} X_{2019} = \left( \begin{matrix} 7.1 &{}49.7 &{}32.9 &{}\dots &{} 39.3 \\ 14.4 &{}7.4 &{}37.4 &{}\dots &{} 96.6 \\ 7.8 &{}19.6 &{}4.4 &{}\dots &{} 58.2 \\ \vdots &{} \vdots &{} \vdots &{}\ddots &{} \vdots \\ 5.6 &{}87 &{}25.3 &{}\dots &{} 0.0 \\ \end{matrix} \right) . \end{aligned}$$

Similarly, we equally constructed $X_{2020}$ and $X_{2021}$ for years 2020 and 2021, respectively. We constructed the grey decision matrix based on Eq. (23):

42 $$\begin{aligned} \otimes X = \left( \begin{matrix} [7.1, 8] &{}[49.7, 53.8] &{}\dots &{} [39.3, 100] \\ [14.4, 15.8] &{}[7.4, 9.1] &{}\dots &{} [56.5, 96.6] \\ [7.2, 8.1] &{}[19.6, 28.2] &{}\dots &{} [53.7, 58.2] \\ \vdots &{} \vdots &{} \ddots &{} \vdots \\ [5.5, 5.7] &{}[82.3, 87]&{}\dots &{} [0, 31] \\ \end{matrix} \right) \end{aligned}$$

Then, we calculated the grey sum ($\otimes X^{\alpha }$), min-max ($\otimes X^{\beta }$), vector ($\otimes X^{\vartheta }$), and max ($\otimes X^{\eta }$) normalization matrix using Eqs. (24), (26), (28), and (29), respectively.

43 $$\begin{aligned} \begin{aligned} \otimes X^{SN} =&\left( \begin{matrix} [0.007, 0.0079] &{}[0.0341, 0.0369] &{}\dots &{} [0.0128, 0.0325] \\ [0.0142, 0.0156] &{}[0.0051, 0.0062] &{}\dots &{} [0.0184, 0.0314] \\ [0.0071, 0.008] &{}[0.0134, 0.0193] &{}\dots &{} [0.0175, 0.0189] \\ \vdots &{} \vdots &{}\ddots &{} \vdots \\ [0.0054, 0.0056] &{}[0.0565, 0.0597] &{}\dots &{} [0, 0.0101] \\ \end{matrix} \right) . \end{aligned} \end{aligned}$$

The normalized grey decision matrices $\otimes X^{\beta }$, $\otimes X^{\vartheta }$, and $\otimes X^{\eta }$ are omitted here. Thus, we obtained the elements of the normalized decision matrix of $\otimes X^*$ using Eq. (31):

44 $$\begin{aligned} \begin{aligned} \otimes X^* =&\left( \begin{matrix} [0.007, 0.08] &{}[0.0336, 0.154] &{}\dots &{} [0.006, 0.1696] \\ [0.0142, 0.158] &{}[0.005, 0.026] &{}\dots &{} [0.0087, 0.1638] \\ [0.0071, 0.081] &{}[0.0132, 0.0807] &{}\dots &{} [0.0083, 0.0987] \\ \vdots &{} \vdots &{}\ddots &{} \vdots \\ [0.0054, 0.057] &{}[0.0556, 0.249] &{}\dots &{} [0, 0.0526] \\ \end{matrix} \right) . \end{aligned} \end{aligned}$$

**Figure 5:**
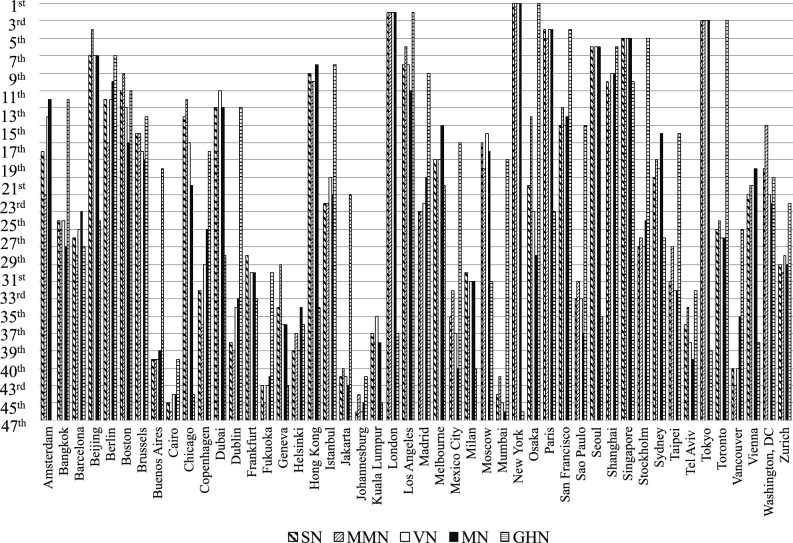
Rankings based on various normalization methods.

We computed the entropy weight using Eq. (13) for the years 2019, 2020, and 2021 to obtain the grey entropy weight using Eq. (14). The grey weights used in this study are given in Eq. (48), the transpose of the grey weight column vector. Please see Table 5 for the complete elements of the matrix (Fig. 5).

45 $$\begin{aligned} W_{2019}= & {} \left( \begin{matrix} 0.0208&0.0115&0.0109&\dots&0.0051 \end{matrix} \right) ^\text {T}, \end{aligned}$$

46 $$\begin{aligned} W_{2020}= & {} \left( \begin{matrix} 0.0206&0.0105&0.0099&\dots&0.0051 \end{matrix} \right) ^\text {T}, \end{aligned}$$

47 $$\begin{aligned} W_{2021}= & {} \left( \begin{matrix} 0.0215&0.0108&0.0039&\dots&0.0051 \end{matrix} \right) ^\text {T}, \end{aligned}$$

48 $$\begin{aligned} \otimes W= & {} \left( \begin{matrix} [1.9288, 2.0091]&[0.9844, 1.0758]&\dots&[0.4733, 0.4799] \end{matrix} \right) ^\text {T}. \end{aligned}$$

**Table 5:** Grey entropy weights for GPCI.

Indicators (*v*)	2019	2020	2021	$\otimes W\times 100^{-1}$	Indicators (*v*)	2019	2020	2021	$\otimes W\times 100^{-1}$
1	2.077	2.0616	2.1474	[1.9288, 2.0091]	36	3.568	3.2819	3.2399	[3.0313, 3.3382]
2	1.1499	1.0521	1.0767	[0.9844, 1.0758]	37	1.7238	1.9829	2.3172	[1.6128, 2.168]
3	1.0917	0.9905	0.385	[0.3602, 1.0214]	38	0.1559	0.1401	0.1408	[0.1311, 0.1459]
4	0.6309	0.5882	0.5398	[0.5051, 0.5903]	39	0.3238	0.3148	0.3668	[0.2946, 0.3432]
5	6.4037	6.4347	6.7562	[5.9914, 6.3212]	40	0.547	0.4124	0.3177	[0.2972, 0.5118]
6	4.7399	4.9399	5.0681	[4.4347, 4.7418]	41	0.5026	0.5037	0.5286	[0.4702, 0.4946]
7	2.1081	2.1393	2.1607	[1.9723, 2.0216]	42	0.5575	0.5502	0.5619	[0.5147, 0.5257]
8	0.667	0.3978	0.3888	[0.3638, 0.6241]	43	0.1777	0.1773	0.2122	[0.1659, 0.1985]
9	1.0275	1.0151	1.0146	[0.9493, 0.9613]	44	0.0977	0.0956	0.0955	[0.0894, 0.0914]
10	0.8527	0.6453	0.7923	[0.6038, 0.7978]	45	0.25	0.2439	0.2401	[0.2247, 0.2339]
11	0.5153	0.6984	0.756	[0.4821, 0.7074]	46	0.5886	0.5634	0.5784	[0.5272, 0.5507]
12	0.9032	0.8628	0.835	[0.7813, 0.8451]	47	0.3963	0.3429	0.4536	[0.3209, 0.4244]
13	0.3387	0.236	0.2286	[0.2139, 0.3169]	48	0.6601	0.6774	0.8134	[0.6176, 0.7611]
14	2.2196	2.2481	2.2195	[2.0766, 2.1034]	49	0.6651	0.6509	0.5868	[0.549, 0.6223]
15	2.5902	2.4555	2.4488	[2.2912, 2.4235]	50	1.0324	0.9129	0.7336	[0.6863, 0.966]
16	3.1489	3.0983	3.1066	[2.8988, 2.9461]	51	0.812	0.8317	0.7181	[0.6718, 0.7781]
17	1.6912	1.5704	1.6697	[1.4693, 1.5824]	52	1.0018	0.5789	0.5189	[0.4854, 0.9373]
18	0.3988	0.4065	0.4464	[0.3731, 0.4177]	53	1.4704	1.4461	1.3106	[1.2262, 1.3757]
19	4.3912	4.318	4.2812	[4.0056, 4.1085]	54	0.9355	0.6836	0.6813	[0.6374, 0.8752]
20	6.2878	5.9898	5.8686	[5.4908, 5.8829]	55	0.2015	0.2366	0.2235	[0.1886, 0.2214]
21	0.6369	2.4749	2.6314	[0.5959, 2.462]	56	0.2107	0.3088	0.3456	[0.1972, 0.3233]
22	4.0838	4.1376	4.1799	[3.8208, 3.9108]	57	0.2754	0.5906	0.5845	[0.2576, 0.5525]
23	0.6601	0.6938	0.6527	[0.6107, 0.6491]	58	0.8852	0.8444	0.8051	[0.7533, 0.8282]
24	4.1151	3.9073	4.0935	[3.6557, 3.8501]	59	0.7277	0.4455	0.3764	[0.3522, 0.6809]
25	4.4636	4.3499	4.2347	[3.962, 4.1762]	60	0.5661	0.5322	0.4259	[0.3985, 0.5296]
26	0.638	0.8983	0.6374	[0.5963, 0.8404]	61	0.992	1.0827	1.0074	[0.9282, 1.013]
27	2.414	2.3626	2.3614	[2.2093, 2.2585]	62	2.0429	2.6161	2.8624	[1.9114, 2.6781]
28	1.5071	1.4181	1.3032	[1.2193, 1.4101]	63	1.5434	1.1515	1.6025	[1.0774, 1.4993]
29	1.9823	1.8819	2.0194	[1.7608, 1.8894]	64	1.0617	1.443	1.0694	[0.9934, 1.3501]
30	1.5195	1.4914	1.4907	[1.3947, 1.4216]	65	1.6214	1.4735	1.5298	[1.3786, 1.517]
31	1.4432	1.3751	1.3259	[1.2405, 1.3503]	66	0.6616	0.6504	0.6517	[0.6086, 0.619]
32	1.3665	1.3831	1.375	[1.2785, 1.294]	67	0.3007	0.5117	0.5644	[0.2814, 0.5281]
33	2.3826	2.3778	2.3328	[2.1826, 2.2292]	68	0.5171	0.2239	0.4387	[0.2095, 0.4838]
34	0.952	0.8492	0.6603	[0.6178, 0.8907]	69	0.3025	0.3258	0.3897	[0.283, 0.3646]
35	0.7201	0.9175	0.7065	[0.661, 0.8584]	70	0.5059	0.506	0.513	[0.4733, 0.4799]

Next, the weighted grey normalized decision matrix is obtained using Eq. (33):

49 $$\otimes D^{*} = \left( {\begin{array}{*{20}c} {[0.0135,0.1607]} & {[0.033,0.1656]} & \ldots & {[0.0029,0.0814]} \\ {[0.0274,0.3174]} & {[0.0049,0.028]} & \ldots & {[0.0041,0.0786]} \\ {[0.0137,0.1627]} & {[0.013,0.0868]} & \ldots & {[0.0039,0.0474]} \\ \vdots & \vdots & \ddots & \vdots \\ {[0.0105,0.1145]} & {[0.0547,0.2678]} & \ldots & {[0,0.0252]} \\ \end{array} } \right)$$

The references are obtained using Eqs. (34) and (35).

50 $$\begin{aligned} \otimes D^+_0= & {} 100^{-1} \times \left( \begin{matrix} 2.0091&1.0758&1.0214&\dots&0.4799 \end{matrix} \right) , \end{aligned}$$

51 $$\begin{aligned} \otimes D^-_0= & {} 100^{-1} \times \left( \begin{matrix} 0.1904&0.0665&0.0043&\dots&0.0068 \end{matrix} \right) . \end{aligned}$$

The difference among the references and the alternatives are obtained using Eqs. (36) and (37).

52 $$\begin{aligned} \Delta ^+= & {} 100^{-1} \times \left( \begin{matrix} 1.8484&{} 0.497&{} 0.5189&{} \dots &{} 0 \\ 1.6917&{} 0.9779&{} 0.4923&{} \dots &{} 0.0163 \\ 1.8464&{} 0.7725&{} 0.5036&{} \dots &{} 0.2006 \\ \vdots &{} \vdots &{} \vdots &{} \ddots &{} \vdots \\ 1.8946&{} 0.1399&{} 0.5842&{} \dots &{} 0.3312 \\ \end{matrix} \right) \end{aligned}$$

53 $$\begin{aligned} \Delta ^-= & {} 100^{-1} \times \left( \begin{matrix} 0.1769&{} 0.0334&{} 0.0025&{} \dots &{} 0.0039 \\ 0.163&{} 0.0616&{} 0.0024&{} \dots &{} 0.0027 \\ 0.1767&{} 0.0534&{} 0.0041&{} \dots &{} 0.0029 \\ \vdots &{} \vdots &{} \vdots &{} \ddots &{} \vdots \\ 0.1799&{} 0.0118&{} 0.0033&{} \dots &{} 0.0068 \\ \end{matrix} \right) \end{aligned}$$

The positive and negative grey relational grades (GRC) are computed using Eqs. (38) and (39):

54 $$\begin{aligned} GRG^+= & {} 100^{-1} \times \left( \begin{matrix} 0.9453&0.9412&0.9414&\dots&0.9408 \end{matrix} \right) ^\text {T} \end{aligned}$$

55 $$\begin{aligned} GRG^-= & {} 100^{-1} \times \left( \begin{matrix} 0.9575&0.9567&0.9560&\dots&0.9559 \end{matrix} \right) ^\text {T} \end{aligned}$$

The ranking scores of the cities are obtained using Eq. (40), which are then ranked.

56 $$\begin{aligned} \begin{aligned} V_i&= 100^{-1} \times \left( \begin{matrix} 0.9514&0.9490&0.9487&\dots&0.9483 \end{matrix} \right) ^\text {T}\\&\approx 14\text {th}\quad 26\text {th}\quad 28\text {th}\quad 7\text {th}\quad 12\text {th}\quad 11\text {th}\quad \dots \quad 30\text {th} )^\text {T}\\ \end{aligned} \end{aligned}$$

**Figure 6:**
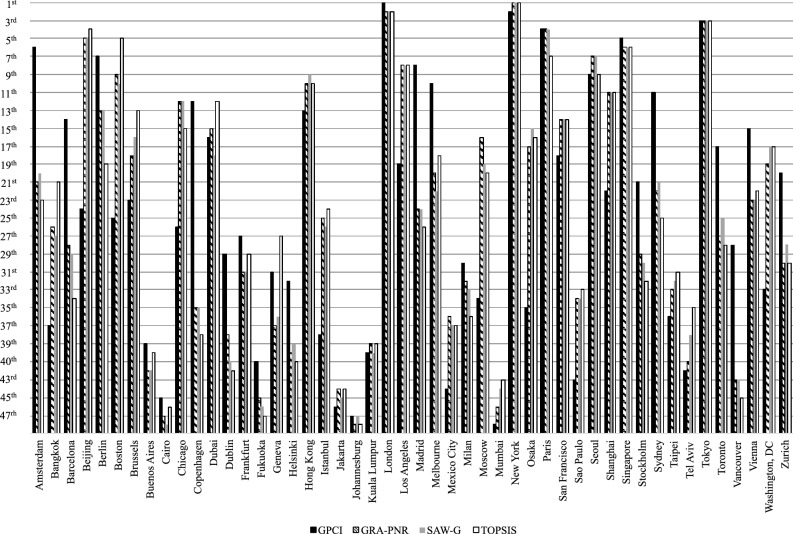
2022 GPCI ranking comparison with rankings produced by grey MCDM methods.

Thus, the ranking of alternatives in descending order is: New York > London > Tokyo > Paris > Singapore > Seoul > Beijing > Los Angeles > Hong Kong > Shanghai > Boston > Berlin > Dubai > Amsterdam > Chicago > San Francisco > Melbourne > Moscow > Brussels > Sydney > Vienna > Osaka > Washington, DC > Madrid > Istanbul > Bangkok > Toronto > Barcelona > Stockholm > Zurich > Copenhagen > Frankfurt > Milan > Taipei > Sao Paulo > Geneva > Dublin > Helsinki > Kuala Lumpur > Mexico City > Vancouver > Buenos Aires > Tel Aviv > Jakarta > Fukuoka > Cairo > Mumbai > Johannesburg. Addtionally, the complete rankings are presented in Fig. 6 and Table 7 (grey entropy weight column) .

### Sensitivity analysis with comparison of approaches

We conducted a sensitivity analysis to show the impact of uncertainty on the ranking of these cities.

#### Time sensitivity

Business decisions must be dynamic to keep up with customer demand; this dynamic characteristic can be captured over time. To understand the effect of time on rankings, we considered the ranking of these cities using data from 2019 to 2021, from 2020 to 2021, and just for 2021 based on GHN, grey entropy weighing method, and GRA-PR. The rankings for these periods are given in Table 6. We observed that the rankings of these periods differ because the considered cities used were not volatile. For example, social infrastructure may degrade over a decade, unlike the stock market, which can change more quickly. The rankings of Boston ($A_{6}$) Chicago ($A_{10}$), and Osaka ($A_{32}$) improved as more data were considered over time to capture uncertainty in the evaluation. In contrast, Helsinki ($A_{17}$) and Shanghai ($A_{37}$) dropped in the rankings uncertainty was considered.

**Table 6:** Period sensitivity.

Cities ($A_i$)	2019 to 2021	2020 to 2021	2021
Amsterdam ($A_{1}$)	$14\text {th}$	$12\text {th}$	$12\text {th}$
Bangkok ($A_{2}$)	$26\text {th}$	$26\text {th}$	$26\text {th}$
Barcelona ($A_{3}$)	$28\text {th}$	$28\text {th}$	$28\text {th}$
Beijing ($A_{4}$)	$7\text {th}$	$7\text {th}$	$7\text {th}$
Berlin ($A_{5}$)	$12\text {th}$	$11\text {th}$	$11\text {th}$
Boston ($A_{6}$)	$11\text {th}$	$13\text {th}$	$14\text {th}$
Brussels ($A_{7}$)	$19\text {th}$	$20\text {th}$	$19\text {th}$
Buenos Aires ($A_{8}$)	$42\text {th}$	$40\text {th}$	$41\text {th}$
Cairo ($A_{9}$)	$46\text {th}$	$46\text {th}$	$46\text {th}$
Chicago ($A_{10}$)	$15\text {th}$	$19\text {th}$	$20\text {th}$
Copenhagen ($A_{11}$)	$31\text {st}$	$30\text {th}$	$30\text {th}$
Dubai ($A_{12}$)	$13\text {th}$	$14\text {th}$	$13\text {th}$
Dublin ($A_{13}$)	$37\text {th}$	$38\text {th}$	$38\text {th}$
Frankfurt ($A_{14}$)	$32\text {nd}$	$32\text {nd}$	$32\text {nd}$
Fukuoka ($A_{15}$)	$45\text {th}$	$44\text {th}$	$44\text {th}$
Geneva ($A_{16}$)	$36\text {th}$	$36\text {th}$	$37\text {th}$
Helsinki ($A_{17}$)	$38\text {th}$	$37\text {th}$	$36\text {th}$
Hong Kong ($A_{18}$)	$9\text {th}$	$8\text {th}$	$9\text {th}$
Istanbul ($A_{19}$)	$25\text {th}$	$23\text {rd}$	$23\text {rd}$
Jakarta ($A_{20}$)	$44\text {th}$	$45\text {th}$	$45\text {th}$
Johannesburg ($A_{21}$)	$48\text {th}$	$47\text {th}$	$47\text {th}$
Kuala Lumpur ($A_{22}$)	$39\text {th}$	$41\text {th}$	$39\text {th}$
London ($A_{23}$)	$2\text {nd}$	$2\text {nd}$	$2\text {nd}$
Los Angeles ($A_{24}$)	$8\text {th}$	$10\text {th}$	$10\text {th}$
Madrid ($A_{25}$)	$24\text {th}$	$22\text {nd}$	$22\text {nd}$
Melbourne ($A_{26}$)	$17\text {th}$	$17\text {th}$	$17\text {th}$
Mexico City ($A_{27}$)	$40\text {th}$	$43\text {th}$	$43\text {th}$
Milan ($A_{28}$)	$33\text {rd}$	$33\text {rd}$	$33\text {rd}$
Moscow ($A_{29}$)	$18\text {th}$	$18\text {th}$	$18\text {th}$
Mumbai ($A_{30}$)	$47\text {th}$	$48\text {th}$	$48\text {th}$
New York ($A_{31}$)	$1\text {st}$	$1\text {st}$	$1\text {st}$
Osaka ($A_{32}$)	$22\text {nd}$	$24\text {th}$	$25\text {th}$
Paris ($A_{33}$)	$4\text {th}$	$4\text {th}$	$4\text {th}$
San Francisco ($A_{34}$)	$16\text {th}$	$15\text {th}$	$15\text {th}$
Sao Paulo ($A_{35}$)	$35\text {th}$	$35\text {th}$	$35\text {th}$
Seoul ($A_{36}$)	$6\text {th}$	$6\text {th}$	$6\text {th}$
Shanghai ($A_{37}$)	$10\text {th}$	$9\text {th}$	$8\text {th}$
Singapore ($A_{38}$)	$5\text {th}$	$5\text {th}$	$5\text {th}$
Stockholm ($A_{39}$)	$29\text {th}$	$29\text {th}$	$29\text {th}$
Sydney ($A_{40}$)	$20\text {th}$	$16\text {th}$	$16\text {th}$
Taipei ($A_{41}$)	$34\text {th}$	$34\text {th}$	$34\text {th}$
Tel Aviv ($A_{42}$)	$43\text {th}$	$42\text {th}$	$42\text {th}$
Tokyo ($A_{43}$)	$3\text {rd}$	$3\text {rd}$	$3\text {rd}$
Toronto ($A_{44}$)	$27\text {th}$	$27\text {th}$	$27\text {th}$
Vancouver ($A_{45}$)	$41\text {th}$	$39\text {th}$	$40\text {th}$
Vienna ($A_{46}$)	$21\text {st}$	$21\text {st}$	$21\text {st}$
Washington, DC ($A_{47}$)	$23\text {rd}$	$25\text {th}$	$24\text {th}$
Zurich ($A_{48}$)	$30\text {th}$	$31\text {st}$	$31\text {st}$

#### Weighting comparison

The GPCI report uses equal weights, as given in Eq. (8). In this study, analysis using equal weight was not considered because decision makers know that the evaluation criteria in ranking an MCDM problem would not have equal degrees of importance, and using equal weights is a poor surrogate for unknown weights^70,71^ (Equal Weight is added for completeness since GPCI used equal weight in 2021 report). The comparison of weighting with a change in rankings ($\Delta$) is presented in Table 7. Different weights usually lead to different rankings. However, considering uncertainty in normalization and performance using the GRA-PNR method, the rankings of Cairo ($A_6$), Hong Kong ($A_{18}$), Paris ($A_{33}$) Seoul ($A_{36}$), Singapore ($A_{38}$), and Tokyo ($A_{43}$) do not change. We found the largest change in ranking for Chicago ($A_{10}$) with a ranking of 28 with equal weights and 15 considering grey entropy weights of the indicators used in the evaluation.

**Table 7:** Difference between equal-weight (EW) rankings and grey entropy (GE).

$A_i$	EW	GE	$\Delta$	$A_i$	EW	GE	$\Delta$
$A_{1}$	$7\text {th}$	$14\text {th}$	7	$A_{25}$	$13\text {th}$	$24\text {th}$	11
$A_{2}$	$37\text {th}$	$26\text {th}$	11	$A_{26}$	$10\text {th}$	$17\text {th}$	7
$A_{3}$	$22\text {nd}$	$28\text {th}$	6	$A_{27}$	$44\text {th}$	$40\text {th}$	4
$A_{4}$	$16\text {th}$	$7\text {th}$	9	$A_{28}$	$35\text {th}$	$33\text {rd}$	2
$A_{5}$	$8\text {th}$	$12\text {th}$	4	$A_{29}$	$25\text {th}$	$18\text {th}$	7
$A_{6}$	$23\text {rd}$	$11\text {th}$	12	$A_{30}$	$48\text {th}$	$47\text {th}$	1
$A_{7}$	$26\text {th}$	$19\text {th}$	7	$A_{31}$	$2\text {nd}$	$1\text {st}$	1
$A_{8}$	$39\text {th}$	$42\text {th}$	3	$A_{32}$	$31\text {st}$	$22\text {nd}$	9
$A_{9}$	$46\text {th}$	$46\text {th}$	0	$A_{33}$	$4\text {th}$	$4\text {th}$	0
$A_{10}$	$28\text {th}$	$15\text {th}$	13	$A_{34}$	$18\text {th}$	$16\text {th}$	2
$A_{11}$	$19\text {th}$	$31\text {st}$	12	$A_{35}$	$41\text {th}$	$35\text {th}$	6
$A_{12}$	$12\text {th}$	$13\text {th}$	1	$A_{36}$	$6\text {th}$	$6\text {th}$	0
$A_{13}$	$36\text {th}$	$37\text {th}$	1	$A_{37}$	$14\text {th}$	$10\text {th}$	4
$A_{14}$	$27\text {th}$	$32\text {nd}$	5	$A_{38}$	$5\text {th}$	$5\text {th}$	0
$A_{15}$	$43\text {th}$	$45\text {th}$	2	$A_{39}$	$21\text {st}$	$29\text {th}$	8
$A_{16}$	$33\text {rd}$	$36\text {th}$	3	$A_{40}$	$11\text {th}$	$20\text {th}$	9
$A_{17}$	$32\text {nd}$	$38\text {th}$	6	$A_{41}$	$38\text {th}$	$34\text {th}$	4
$A_{18}$	$9\text {th}$	$9\text {th}$	0	$A_{42}$	$42\text {th}$	$43\text {th}$	1
$A_{19}$	$34\text {th}$	$25\text {th}$	9	$A_{43}$	$3\text {rd}$	$3\text {rd}$	0
$A_{20}$	$45\text {th}$	$44\text {th}$	1	$A_{44}$	$24\text {th}$	$27\text {th}$	3
$A_{21}$	$47\text {th}$	$48\text {th}$	1	$A_{45}$	$30\text {th}$	$41\text {th}$	11
$A_{22}$	$40\text {th}$	$39\text {th}$	1	$A_{46}$	$17\text {th}$	$21\text {st}$	4
$A_{23}$	$1\text {st}$	$2\text {nd}$	1	$A_{47}$	$29\text {th}$	$23\text {rd}$	6
$A_{24}$	$15\text {th}$	$8\text {th}$	7	$A_{48}$	$20\text {th}$	$30\text {th}$	10

#### Normalization comparison

In the comparison of rankings, GSN, GMMN, GVN, GMN, and GHN in “Uncertainty in Normalizing Decision Table” Section were independently used in ranking the cities using the grey decision-matrix in Eq. (42), grey entropy weight in Eq. (48), and GRA-PR. Figure 5 shows the rankings achieved using the various normalizations. The rankings of New York, London, and Tokyo as first, second, and third, respectively, are consistent across all normalizations. Notably, the GPCI for the year 2022 ranked London in first and New York in second. More importantly, the rankings of Berlin, Boston, Chicago, Copenhagen, Moscow, and Tel Aviv all differ using the five different types of normalization, which confirms the uncertainty in the rankings using different normalization methods.

The rankings of the of the top cities are relatively stable for the first- to fourth-placed cites.

#### Evaluation comparison

**SAW with Grey Relations** The classical simple additive weighting (SAW) method was extended to GST by Zavadskas^68^ and is called simple additive weighting with grey relations (SAW-G). The main idea of SAW-G is to compute the weighted grey decision matrix and aggregate the criteria for the alternative, and the weighted values of the alternatives are ranked. The steps used are as follows:

1. Establish the evaluation criteria, see Table 3.
2. Construct the grey decision matrix using Eq. (23).
3. Obtain the GSN of the grey decision matrix using Eq. (23) and given in Eq. (43).
4. Obtain the criteria weights, *W*. The grey entropy weights in Eq. (48) are used.
5. Compute the weighted normalized decision matrix using Eq. (33).
6. Calculate the optimality criteria, $L_i$, using Eq. (57).
  57 $$\begin{aligned} L_i= & {} \frac{1}{n}\sum \limits _{j=1}^m{\frac{\underline{d^*_{ij}}+\overline{d^*_{ij}}}{2}} \end{aligned}$$
  58 $$\begin{aligned} L_i= & {} \left( \begin{matrix} 0.0348&0.0278&0.0258&0.0629&\dots&0.0263 \end{matrix} \right) ^\texttt {T}\,\nonumber \\\approx & {} \left( \begin{matrix} 20{\textrm{th}}&27{\textrm{th}}&29{\textrm{th}}&5{\textrm{th}}&\dots&28{\textrm{th}} \end{matrix} \right) ^T. \end{aligned}$$

$\therefore$    New York > London > Tokyo > Paris > Beijing > Singapore > Seoul > Los Angeles > Hong Kong > Boston > Shanghai > Chicago > Berlin > San Francisco > Osaka > Brussels > Washington, DC > Dubai > Moscow > Amsterdam > Sydney > Melbourne > Vienna > Madrid > Toronto > Istanbul > Bangkok > Zurich > Barcelona > Stockholm > Frankfurt > Taipei > Milan > Sao Paulo > Copenhagen > Geneva > Mexico City > Tel Aviv > Helsinki > Kuala Lumpur > Dublin > Buenos Aires > Vancouver > Mumbai > Jakarta > Fukuoka > Johannesburg > Cairo. Figure 6 shows the complete ranking using SAW-G in comparison with the GPCI for the year 2022 and the TOPSIS-G method.

*TOPSIS with grey values* Lin et al.^72^ extended the TOPSIS method using GST; the steps are as follows:

1. Construct the the grey decision matrix base on Eq. (12) and given in Eq. (42)
2. Normalize the grey decision matrix using Eq. (28), which is based on VN.
3. Calculate the weighted normalized grey decision matrix using Eq. (33).
4. Compute both the positive and negative ideal solutions.
  - The positive ideal solution is:
    59 $$\begin{aligned} \begin{aligned} D_{}^{+}&=\left[ d_{1}^{\text {+}}\quad d_{2}^{\text {+}}\quad d_{3}^{\text {+}} \cdots d_{48}^{+} \right] \\ {}&=\left[ 0.7159 \quad 0.3079 \quad 0.2252 \quad \cdots \quad 0.0814 \right] \end{aligned} \end{aligned}$$
    where $$d_{j}^{+}=$$ $$\left\{ \left( \underset{1\le i\le 48}{\mathop {\max }}\,\overline{ {d}_{ij}^{*}} \left| \right. j \in J \right) , \left( \underset{1\le i\le 48}{\mathop {min }}\, {d}_{ij}^{*} \left| \right. j \in J \right) \left| \right. i \in n \right\}$$
  - The negative ideal solution is:
    60 $$\begin{aligned} \begin{aligned} D_{}^{-}&=\left[ d_{1}^{\text {-}}\quad d_{2}^{\text {-}}\quad d_{3}^{\text {-}}\quad \cdots \quad d_{48}^{-} \right] \\ {}&=\left[ 0\quad 0\quad 0\quad \cdots \quad 0 \right] , \end{aligned} \end{aligned}$$
    where $$d_{j}^{-}=$$ $$\left\{ \left( \underset{1\le i\le 48}{\mathop {\min }}\, {d}_{ij}^{*} \left| \right. j \in J \right) , \left( \underset{1\le i\le 48}{\mathop {max}}\,\overline{{d}_{ij}^{*}}\left| \right. j \in J \right) \left| \right. i \in n \right\}$$
5. Calculate the gap from the ideal solution to obtain the positive and negative distances using Eqs. (61) and (62), respectively.
  - The positive ideal points are:
    61 $$D^{ + } = (\begin{array}{*{20}l} {D_{1}^{ + } } \hfill & {D_{2}^{ + } } \hfill & {D_{3}^{ + } } \hfill & \cdots \hfill & {D_{{48}}^{ + } } \hfill \\ \end{array} )^{{\text{T}}} = \left( {\begin{array}{*{20}l} {1.0931} \hfill & {1.1573} \hfill & {0.7602} \hfill & \cdots \hfill & {0.7711} \hfill \\ \end{array} } \right)^{{\text{T}}}$$
    where $d_{ij}^{+}\sqrt{{{\left( \dfrac{1}{2} \sum \limits _{i=1}^{n}{{{\left( \left| \underline{d_{ij}^{*}}- {d_{_{j}}^{+}} \right| ^{2 }+ \left| \overline{d_{ij}^{*}}- {d_{_{j}}^{+}} \right| ^{2 } \right) }}} \right) }}}$ is the Euclidean distance, and the aggregated criteria are $D^+_{i}=d^{+}_{i1} + d^{+}_{i2} +\cdots + d^{+}_{i70}$
  - The negative ideal points are:
    62 $$D^{ - } = \left( {\begin{array}{*{20}c} {D_{1}^{ - } } & {D_{2}^{ - } } & {D_{3}^{ - } \cdots D_{{48}}^{ - } } \\ \end{array} } \right)^{T} = \left( {\begin{array}{*{20}c} {7.1437\quad 7.3281\quad 7.3263 \cdots 7.2365} \\ \end{array} } \right)^{T}$$
    where $D_{i}^{-}=$ $\sqrt{{{\left( \dfrac{1}{2} \sum \limits _{i=1}^{n}{{{\left( \left| \underline{d_{ij}^{*}}- {d_{_{j}}^{-}} \right| ^{2 }+ \left| \overline{d_{ij}^{*}}- {d_{_{j}}^{-}} \right| ^{2 } \right) }}} \right) }}}$ is the Euclidean distance, and the aggregated criteria are $D^-_{i}=d^{-}_{i1} + d^{-}_{i2} +\cdots + d^{-}_{i70}$
6. Compute the similarities to the positive ideal solution. The similarities of the SC to the positive ideal alternative are computed using Eq. (63):
  63 $$\begin{aligned} \begin{aligned} \begin{aligned} T =&\left( \begin{matrix} 0.8673&0.8636&0.9060&\cdots&0.9037 \end{matrix} \right) ^\text {T}\\ \approx&\left( \begin{matrix} 23\text {rd}&21\text {st}&34\text {th} \cdots 30\text {th} \end{matrix} \right) ^\text {T}, \end{aligned} \end{aligned} \end{aligned}$$
  where $$T_i = \frac{ D_{i}^{-}}{ D_{i}^{-}+ D_{i}^{+}}$$.

$\therefore \quad$ New York > London > Tokyo > Beijing > Boston > Singapore > Paris > Los Angeles > Seoul > Hong Kong > Shanghai > Dubai > Brussels > San Francisco > Chicago > Osaka > Washington, DC > Melbourne > Berlin > Moscow > Bangkok > Vienna > Amsterdam > Istanbul > Sydney > Madrid > Geneva > Toronto > Frankfurt > Zurich > Taipei > Stockholm > Sao Paulo > Barcelona > Tel Aviv > Milan > Mexico City > Copenhagen > Kuala Lumpur > Buenos Aires > Helsinki > Dublin > Mumbai > Jakarta > Vancouver > Cairo > Fukuoka > Johannesburg. Figure 6 shows the 2021 GPCI rankings in comparison with those produced by GRA-PNR, SAW-G, and TOPSIS-G. Although the GPCI ranked London first, after considering uncertainty, the three methods based on GST that accounted for uncertainty also ranked New York first. Additionally, all methods ranked Tokyo in third.

Different ranking methods produce different rankings, but the ranking should be highly correlated for decision making. Therefore, the Spearman’s rho ($\rho$) and Kendall’s tau ($\tau$) correlation are presented in Table 8. GRA-PNR, SAW-G, and TOPSIS-G are highly correlated. The highest correlation among the methods is between GRA-PNR and SAW-G; the GPCI for the year 2022 showed the weakest correlation with TOPSIS-G.

**Table 8:** Correlation of MCDM methods.

	GPCI	GRA-PNR	SAW-G	TOPSIS-G
GPCI	1.0000	0.6365	0.5621	0.5000
GRA-PNR	(0.8089)	1.0000	0.9078	0.8316
SAW-G	(0.7454)	(0.9830)	1.0000	0.8848
TOPSIS-G	(0.6769)	(0.9607)	(0.9785)	1.0000

Kendall-$$\tau$$ ( Spearman-$$\rho$$)

## Conclusions

The aim of decision making in management is to achieve organizational goals, and MCDM methods provide a systematic procedure for selecting the appropriate alternative as solution to a decision problem. Each previous normalization method does not account for uncertainty, which is not obtainable in the real world. In other words, there is uncertainty. Also, the values normalization methods result in different rankings and introduce uncertainty. Deductively, a technique that shifts from an “ideal situation” to a “real situation” is better considering the premise of the circumstances. In real situations, uncertainties exist, as exemplified by the COVID-19 pandemic, and should be captured when evaluating alternatives. Unfortunately, we only have partial information about the natural world because it is random and dynamic, as exemplified by continued forecasting and predicting research. The GST deals with a system with incomplete information, partial information. This paper presented a new layer of uncertainty, specifically uncertainty in normalization. We combined the four approaches for normalization in the literature, SN, MMN, VN, and MN, as a hybrid approach called grey hybrid normalization (GHN). Also, the period-based entropy weighting method was proposed, and the GRA-PNR over a period was applied in this study. Additionally, uncertainty from the performance values of the criteria and weighting was captured in ranking 48 cities from around the world. In contrast to London being the top ranking city, as reported by the GPCI, we ranked New York as first after considering uncertainty as given in Eq. (56). This result was confirmed using SAW-G and TOPSIS-G methods as giving in Eqs. (58) and (63), respectively.

Our findings have some managerial implications. Although a business would want to be located in a city that will facilitate the company’s efforts to increase sales, create a more positive image, or reduce costs, other factors must be considered that are specific to their operation, such as the company’s proximity to raw materials, employment, competitors, infrastructure, and finance. For example, an oil and gas company would have an office in an oil field, and a farmer would need to be close to land with suitable soil. Being close to raw material reduces the transportation cost, which adds to the production cost. As another example, a high-technology business would want to be located where employees have the appropriate skills, such as close to universities and colleges, which would translate to higher innovation, which translates to higher sales. Factories would want to be located in areas with appropriate labor resources a high employment rate. Higher employment can translate to lower wages, which reduces the cost of running the business. In some cases, on the one hand, the best city may not be suitable: a business may need to be located close to its competitors or the business will fail. For instance, a perfume manufacturer may want to be located close to famous brands, positioning itself as an alternative that provides equally good fragrance. The GPCI captured the infrastructure of the evaluated cities; however, the proximity to target market needs to be considered in ranking cities for a particular business. Additionally, online businesses can be setup anywhere, but being close to a courier and logistic company would be advantageous for online store to pass on those savings to customers. More importantly, coupling, specificity, and formalization are other areas to be considered in the location of the manufacturing industry^73^.

One known limitation of this paper is it focuses on the normalization layer (Layer 2) in the decision-making process. Actually this is intentional so that other layers are controlled by not introducing new methods in Layers 1, 3 and 4 (Fig. 4) since varying any of these Layers can undoubtedly change the rankings of the alternatives. Thus, the paper did not set out to propose new MCDM methods for measuring performance value, weighting and evaluation. For this reason, this paper did not use any subjective methods to maintain its objectivity. Specifically, the performance valves of cities presented in this paper are commonly accepted and reported measuring techniques such as gross domestic product (GDP). Also, the new variation of grey entropy weight (i.e. period-based grey entropy weight) is used even when a hybrid of subjective and objective weights would provide more balanced weights. Group decision-making is not presented in this paper for this same reason. Regardless, capturing uncertainty in decision-making increases computational complexity, and further research is needed to propose a less complex and efficient approach.

Additional limitation of this study is only ranking the cities without tailoring the ranking to the EV industry. The case presented in this paper is a response to the CEO of an EV company in China requesting that all uncertainty in the rankings provided by the GPCI team be accounted for. In some other cases, on the other hand, the best city may not be suitable: a business may need to be located far away from competitors or the business will fail. For example, if a newer Chinese car manufacturing company tries to locate its factory close to that of Toyota in Japan, the managerial implication is that the ranking of location for a business should not be generic, but tailored the a particular business. This opens new direction for future research, which may involve incorporating the subjective weights assigned by the managers of the company and having criteria for measuring their competitors at every site. Lastly, a multi-national business is profitable after accounting for all international costs, which includes the additional cost of expatriate compensation and benefit^74^. Therefore, further analysis can be done to determine the profitability of business decisions under uncertainty, which is beyond location selection.

## Data Availability

Data provided by Global Power City Index (GPCI) of The Mori Memorial Foundation is freely available to the public for download at http://www.mori-m-foundation.or.jp. Moses Olabhele Esangbedo can be contacted for more details, moses@xzit.edu.cn.
