# Author Correction: Grey hybrid normalization with period based entropy weighting and relational analysis for cities rankings

**DOI:** 10.1038/s41598-023-43335-z

**Published:** 2023-09-27

**Authors:** Moses Olabhele Esangbedo, Jieyun Wei

**Affiliations:** School of Management Engineering, Xuzhou University of Technology, No. 2 Lishui Road, Yunlong District, Xuzhou, 221018 China

Correction to: *Scientific Reports* 10.1038/s41598-023-40954-4, published online 23 August 2023

The original version of this Article contained an error in the Funding section.

“This paper is supported by the National Social Science Foundation of China Project Number: 21JBY179.”

now reads:

“This paper is supported by the National Social Science Foundation of China Project Number: 21BJY179.”

The original Article has been corrected.

